# Editorial of Special Issue “Nutrition and Critical Illness”

**DOI:** 10.3390/nu16213640

**Published:** 2024-10-26

**Authors:** George Briassoulis, Stavroula Ilia, Panagiotis Briassoulis

**Affiliations:** 1 Postgraduate Program “Emergency and Intensive Care in Children Adolescents and Young Adults”, School of Medicine, University of Crete, 71003 Heraklion, Greece; stavroula.ilia@uoc.gr; 2 Pediatric Intensive Care Unit, University Hospital, School of Medicine, University of Crete, 71110 Heraklion, Greece; 3 Second Department of Anaesthesiology, Attikon University Hospital, School of Medicine, National and Kapodistrian University of Athens, 12461 Athens, Greece; briaspan@med.uoa.gr

## 1. Introduction

Managing nutrition therapy in critically ill patients is complex due to the ongoing inflammation, catabolic stress, and changing metabolic demands that occur throughout an illness. Intensive Care Unit (ICU) patients are a diverse population, and it is impossible to recommend a one-size-fits-all approach. Nutritional strategies must account for each patient’s diagnosis, the phase of illness (early, stabilization, recovery, rehabilitation), and any complications [1]. Nutritional assessment should be conducted at ICU admission and monitored throughout the stay. Further studies are needed to establish reliable nutrition monitoring indicators to evaluate patients’ responses to nutritional interventions [2]. Phase angle (PhA), measured using bioelectrical impedance analysis (BIA), is a valuable tool in clinical nutrition assessment. A low PhA is linked to prolonged ICU stays and higher mortality risk [3]. A prospective validation study indicated that the ultrasound measurement of femoral quadriceps muscle thickness performs similarly to thigh CT in assessing muscle mass in critically ill patients [4].

Enteral nutrition (EN) is preferred over parenteral nutrition (PN), although recent studies have shown no significant inferiority of PN [5]. However, intragastric EN can be safely administered even during prone positioning and extracorporeal membrane oxygenation. Early EN initiation is encouraged, but there is no evidence supporting high caloric intake during the acute phase of critical illness. Providing energy delivery close to 70% of the estimated energy requirements during the first week of an ICU stay was linked to lower 28-day mortality in mechanically ventilated critically ill patients, particularly those with high nutritional risk at admission [6]. Higher protein delivery, compared to lower intake, does not seem to impact clinical outcomes in the general critically ill population, but may be associated with increased mortality rates in patients with acute kidney injury (AKI) [7]. In cases where EN is insufficient, withholding supplemental PN during the first week in ICU can be considered [1,8,9]. While current guidelines should be followed for micronutrient supplementation in critically ill patients, and specific immune nutrients have shown promising results [10], there is no clear benefit to using pharmaconutrition and there are potential risks [5].

Estimating energy requirements in critically ill children is often inaccurate, leading to the risks of overfeeding or underfeeding. Overfed children tend to have longer ICU stays compared to those appropriately fed or underfed [11]. Therefore, the accurate estimation of energy needs, with a focus on optimizing protein intake, is critical. Where possible, indirect calorimetry (IC) should be used, alongside the careful use of predictive equations and increased monitoring to avoid unintentional misfeeding. In adult studies, IC-measured resting energy expenditure (mREE) often reveals hypo- or hypermetabolism, and estimated REE (eREE) calculations frequently show poor correlation with actual values [12]. Gastrointestinal (GI) dysfunction frequently occurs in critically ill patients but is often underrecognized due to the absence of standardized diagnostic and treatment protocols [13]. Interruptions in enteral nutrition (EN) are also common in the ICU, particularly in children with higher Pediatric Risk of Mortality (PRISM) scores, prolonged ICU admissions, and cardiac conditions, leading to reduced intake of calories and protein [14]. Moreover, most large-scale feeding studies have primarily concentrated on interventions during the initial week of critical illness, creating a gap in understanding the impact of various feeding strategies beyond the acute phase and after ICU discharge [15].

Overall, the management of EN in critically ill patients requires a structured and continuously updated approach that involves all ICU professionals. Ongoing research is vital to refine nutritional strategies to improve patient outcomes. This Special Issue of *Nutrients*, containing multifaceted original research articles and reviews, offers new insights into nutritional evaluation during both the acute and recovery phases of critical illness, proposing adjustments to nutritional strategies and feeding protocols to enhance the effectiveness of therapy for these patients.

## 2. An Overview of Published Articles

The Nutrition Risk in Critically Ill (NUTRIC) score has emerged as a valuable tool for assessing the nutritional risk of Intensive Care Unit (ICU) patients. A secondary analysis of sepsis registry data demonstrated the score’s effectiveness in predicting mortality (AUC 0.833, *p* < 0.001) with an optimal cut-off of 6 points (contribution 1). Additionally, the score correlated with an increased nursing workload, higher need for vasopressor support (98% vs. 82%), mechanical ventilation (99% vs. 87%), renal replacement therapy (54% vs. 26%), steroid use (68% vs. 31%), and blood product administration (60% vs. 43%). A separate retrospective study focusing on ICU patients admitted after cardiac arrest found that higher BMI increased the hazard ratio for in-hospital mortality (contribution 2). However, neither the body mass index (BMI) nor the Nutritional Risk Score (NRS 2002) significantly impacted mortality odds.

The timing of initiating enteral nutrition (EN) in critically ill patients remains debated. A systematic review reported no significant difference in mortality between early and delayed EN in sepsis patients (contribution 3). The risk ratio for short-term mortality across three RCTs was insignificant, and the mean difference in ICU length of stay from two RCTs was −2.91 days and −1.00 days, respectively. However, the evidence is weak due to inconsistent definitions, study heterogeneity, risk of bias, and poor study methodologies. In the era of green ICU research, data from 54,830 adults (aged ≥19 years) in the Third National Health and Nutrition Examination Survey (NHANES) and NHANES 1999–2014, linked with mortality data through 2015, revealed no significant associations between dairy consumption and mortality risk from all causes or cancer when comparing the lowest and highest quartiles of intake. However, dairy consumption was linked to a 26% lower risk of heart disease mortality, with those in the highest quartile of consumption showing a hazard ratio of 0.74 (CI: 0.54–1.01; *p* = 0.05) compared to the lowest quartile (contribution 4). Further analysis by age group found that dairy intake was associated with a 39% and 31% reduced risk of heart disease mortality in adults aged 51–70 and those aged ≥51, respectively.

Gastrointestinal dysfunction often impedes effective EN, resulting in immune dysregulation and systemic inflammation. Gastrointestinal function evaluation can be achieved through biomarker analysis and functional assessments, including tests for epithelial barrier integrity and gastrointestinal motility disorders (contribution 5). A retrospective analysis of data from 1584 ICU patients receiving EN evaluated the prognostic significance of enteral feeding intolerance markers during the early stages of ICU care. It used a machine learning approach to predict early EN failure. The results highlighted gastric residual volume as a key predictor of survival in critically ill patients, alongside factors such as APACHE II scores, age, SOFA scores, and inadequate enteral feeding by day 3 (contribution 6). The machine learning model emphasized the role of enteral feeding intolerance markers in predicting poor 90-day outcomes and early EN failure, supporting the early identification of patients at risk. In sepsis, alterations in biomarkers such as citrulline and intestinal fatty acid binding protein may reflect intestinal permeability and damage changes. A single-center, prospective observational study involving patients with sepsis and septic shock found that citrulline levels were lower in the sepsis group compared to the non-sepsis group, particularly among those with septic shock. Patients with a gastrointestinal failure score (AGI score III) had lower citrulline levels than those with AGI scores I/II, and higher AGI scores were linked to increased 28-day mortality (*p* = 0.038). These findings suggest that citrulline measurements, combined with AGI scoring, may be valuable for monitoring gastrointestinal function and integrity in sepsis patients (contribution 7).

Despite these challenges, many EN interruptions in critically ill patients are unjustified, leading to significant deficits in calories, protein, and antioxidants. A prospective study of ICU patients admitted for over 48 h found that the median duration of EN interruptions was 5.2 (3.4–7.4) hours per day (contribution 8). Gastric residual volume (GRV) monitoring—a controversial practice—was the leading cause of these interruptions. Over half of the patients received less than 65% of their total energy needs, and the median daily protein intake was only 0.43 ± 0.3 g/kg/day, far below the ESPEN recommendation of 1.3 g/kg/day for adjusted body weight (*p* < 0.001). Additionally, the intake of micronutrients and antioxidants (arginine, selenium, zinc, vitamins) was significantly lower than the dietary reference intake (*p* < 0.01).

Parenteral nutrition (PN) is essential for preventing malnutrition when EN cannot be utilized. Findings from a multicenter prospective observational study indicated that the early administration of PN can be safe and effective in delivering adequate nutrition (contribution 9). Combining EN with PN, when feasible, may help meet protein needs, particularly in critically ill patients who start with PN and are anticipated to have prolonged stays in the ICU. Providing sufficient nutrition for preterm infants is essential for supporting growth and promoting optimal neurodevelopment in later stages. A retrospective study evaluated how energy and macronutrient intake affect growth velocity and outcomes, while also considering variations within the diverse preterm population (contribution 10). The findings indicated that achieving an early positive energy and protein balance, following ESPGHAN guidelines, is vital for ensuring healthy postnatal growth and preventing extrauterine growth restriction, which is relatively common among preterm infants.

The adequacy of nutritional intake in critically ill survivors after hospital discharge remains uncertain. A study in this SI assessed energy and protein intake at 1, 3, and 12 months (M1, M3, M12) following an ICU stay of 7 days or longer. At these time points, energy intake reached 73.2%, 79.3%, and 82.7% of the recommended targets for healthy individuals, respectively (*p* = 0.074). Protein intake was below 0.8 g/kg/day in 36.7%, 25.8%, and 13.3% of patients at M1, M3, and M12, respectively (*p* = 0.018), representing 67.9%, 68.5%, and 71.7% of the post-ICU recovery targets (*p* = 0.138). Overall, in this post-ICU cohort, energy and protein intake remained below recommended levels for up to a year after discharge, which is critical for ICU survivors during recovery (contribution 11). Research reports reduced physical capacity for up to five years post ICU. “ICU-acquired weakness” involves a complex combination of muscle atrophy, disrupted myofibrillar protein homeostasis, altered muscle composition, impaired regeneration, issues with excitation–contraction coupling, and acquired mitochondrial dysfunction. A prospective observational study examining the mid-term evolution of the acylcarnitine (AC) profile in patients who stayed in the ICU for seven days or more revealed a significant reduction in free carnitine (C0) and short-chain AC levels (*p* < 0.001), along with an increase in the AC/C0 ratio (*p* = 0.001) at three months post discharge (contribution 12).

Significant metabolic disturbances have been observed in critically ill patients. The acylcarnitine (AC) profile in ICU survivors shows elevated short-chain derivatives compared to normal reference values. In an observational study, 50 cardiac surgery (CS) patients (SAPS II score of 23) who survived a 2-day ICU stay were compared to 85 patients (SAPS II score of 36, *p* < 0.001) who survived a longer ICU stay of 11 days. Neither group showed carnitine deficiency, and their total AC/C0 (free carnitine) ratios were similar. A ratio greater than 0.4, indicating impaired mitochondrial metabolism, did not differ between the groups. Both groups exhibited elevated long-chain ACs, with a greater increase in the CS group, while short-chain ACs were higher in the ICU group (contribution 13). Older cancer patients face a heightened risk of sarcopenia. Data from the NutriAgeCancer French nationwide study were used to analyze the prevalence of four sarcopenia-related criteria: case finding, assessment, diagnosis, and severity determination. The prevalence of abnormal strength, assistance with walking, difficulty rising from a chair, climbing stairs, and falls (SARC-F); low hand-grip strength (HGS); reduced arm circumference (AC); poor physical performance (PP); sarcopenia; and severe sarcopenia ranged from 11.7% to 44.7%. Abnormal SARC-F, low HGS, sarcopenia, and severe sarcopenia were linked to increased 6-month mortality in patients with metastases, with adjusted hazard ratios of between 2.72 and 6.41 (contribution 14).

Indirect calorimetry (IC) is generally preferred for assessing resting energy expenditure (mREE) over predictive equations (eREE). In a retrospective observational study, the median mREE was notably higher in critically ill, mechanically ventilated patients aged 75 and older (1457 kcal/day) compared to a healthy control group matched for age, gender, and body mass index (1351 kcal/day). The accuracy of predictive equations was low, ranging from 21% to 49% (contribution 15). The accuracy of sixteen predictive equations for estimating resting energy expenditure (eREE) was also assessed in comparison to measured resting energy expenditure using indirect calorimetry (mREE) in 153 critically ill children. The calculated eREE either significantly underestimated (median 606, IQR 512–784 kcal/day) or overestimated (1126.6, IQR 929–1340 kcal/day) mREE, which had a median of 928.3 kcal/day (IQR 651–1239 kcal/day). These discrepancies led to substantial biases ranging from −342 to 592 kcal (95% limits of agreement: −1107 to 1380 kcal/day) and high coefficients of variation (up to 1242%). The clinically acceptable accuracy rate of ±10% was achieved in only 6.5% to 24.2% of cases, with inaccuracies varying between −31% and +71.5% of the patient’s actual energy requirements, emphasizing the need for the IC measurement of REE in situations where precise energy assessment is crucial (contribution 16). In the same vein, an observational study was conducted to measure ICU survivors’ mREE during post-ICU hospitalization and compare these measurements with estimates from eREE. In this cohort of patients who had survived an extended ICU stay, mREE was approximately 22–23 kcal/kg/day. However, none of the predictive equations tested was accurate in estimating the energy expenditure measured by indirect calorimetry during the post-ICU hospitalization period (contribution 17).

Due to the limited availability of IC, alternative methods for predicting REE based on carbon dioxide production (VCO_2_) measurements (REEVCO_2_) have been proposed as a substitute for IC-derived REE (mREE). A study assessing the validity of REEVCO_2_ as a substitute for REEIC in mechanically ventilated children found that REEVCO_2_ is not a suitable alternative, regardless of the patient’s metabolic, anthropometric, or clinical status (contribution 18). The study reported a calculated REEVCO_2_, using a respiratory quotient (RQ) of 0.89, with a mean bias of −72.7 kcal/day (95% limits of agreement: −321.7 to 176.3 kcal/day) and a high coefficient of variation (174.7%), with 51.4% of the results falling outside the ±10% accuracy range. Adjusting REEVCO_2_ using RQ values of 0.80 or 0.85 did not improve its accuracy. In contrast, a reduction in VO_2_ in patients with a left ventricular assist device (LVAD) and a pulmonary artery catheter was found to predict in-hospital, 1-year, and 6-year survival, with the highest area under the curve (0.77, 95% CI: 0.6–0.9; *p* = 0.0004) and a cut-off value of 210 mL/min, associated with a hazard ratio of 5.1. DO_2_ was significantly decreased on days 2 and 3 (*p* = 0.007 and *p* = 0.003), suggesting that perioperative and intensive care should prioritize restoring microcirculatory perfusion and mitochondrial function (contribution 19).

Immune-enhancing nutrition, or “pharmaconutrition”, refers to the use of specialized nutrients, including glutamine, alanine, omega-3 fatty acids, and vitamins, that help regulate the body’s response to illness and injury. Agreement is still lacking about the value of individual immune-modulating substrates for critically ill patients. A prospective study involving overweight COVID-19 patients found that immune nutrition effectively prevented the onset of malnutrition and led to a significant reduction in inflammatory markers (contribution 20). When compared to a historical control group that did not receive immune nutrition, the study observed a notable decrease in inflammatory markers (*p* < 0.05), while body mass index (BMI) and phase angle (PA) remained stable without deterioration. An experimental study investigated the effects of lipopolysaccharide (LPS) 1 μg/mL, heat shock (HS) induction at 43 °C, and glutamine (10 mM 1 h before or after stimulation) on heat shock protein-90α (HSP90α) and cytokine responses in an ex vivo model using peripheral blood mononuclear cells (PBMCs). In sepsis, glutamine did not affect the LPS or HS-induced HSP90α mRNA and monocyte HSP90α protein levels. However, in trauma-induced systemic inflammatory response syndrome (SIRS), glutamine administered before LPS reduced monocyte HSP90α, while its administration after HS increased it (*p* = 0.018). In sepsis, glutamine reduced lymphocyte HSP90α expression when given before or after LPS (*p* = 0.049) and before HS (*p* = 0.018) but increased it when administered after HS in SIRS (*p* = 0.003) (contribution 21). Regarding cytokine responses, glutamine significantly enhanced LPS-induced monocyte chemoattractant protein-1 (MCP-1) at 48 h in healthy individuals (*p* = 0.011), SIRS (*p* < 0.001), and sepsis (*p* = 0.006). The study concluded that glutamine (10 mM), whether administered before or after LPS and HS, has varied and unpredictable effects on monocyte and lymphocyte HSP90α in sepsis and SIRS. Another prospective analytical study was carried out on critically ill patients with SIRS after a seven-day stay in the ICU. The study found that both 25-Hydroxyvitamin D (25-OH-D) and 25-OH-D3 levels were significantly correlated with erythrocyte zinc (Zn) concentrations during follow-up (*p* = 0.046 and *p* = 0.011, respectively) (contribution 22). Additionally, a relationship between erythrocyte and plasma zinc levels was observed at the same follow-up point. The study also identified various disturbances in phosphorus and calcium metabolism, indicating a connection between changes in 25-OH-D3 levels and parathyroid hormone (*p* = 0.019) as well as phosphorus levels (*p* = 0.005).

A randomized controlled trial examined the clinical and biochemical impacts of N-acetylcysteine (NAC) administration in critically ill COVID-19 patients. NAC, given through a continuous infusion with an initial loading dose followed by a maintenance dose, was found to prevent the decline in glutathione levels and improve both the clinical and inflammatory responses in severely ill COVID-19 patients compared to the control group (contribution 23). In contrast, certain nutrients can trigger critical illnesses in susceptible individuals. Favism, a hemolytic condition, occurs in people with glucose-6-phosphate dehydrogenase (G6PD) deficiency after consuming fava beans. Some symptoms are age-specific, such as death in infants, visual impairment in children, systolic murmurs in infants, children, and adolescents, and renal failure in adults (contribution 24). The condition tends to be more severe in young children and pregnant women. While the symptoms are usually self-limiting and do not cause lasting damage, hospitalization and blood transfusions are often necessary.

## 3. Conclusions

This collection of articles on nutrition in critical illness showcases a wide array of research, highlighting the depth and complexity of the field. The diversity of methodologies used, from experimental ex vivo studies focusing on intracellular biology to prospective and retrospective observational studies and reviews, reflects the ongoing exploration of nutritional challenges in critical care. While nutritional support is widely recognized as crucial for the recovery of critically ill patients, the lack of methodologically rigorous trials has led to the absence of clear, evidence-based guidelines, resulting in varied practices in ICUs across the globe. Despite extensive research over the years, several aspects of nutrition, such as the ideal timing, dosage, and composition of energy and macronutrient intake, as well as the role of micronutrients, remain contentious due to the considerable heterogeneity in study designs and findings [16]. These inconsistencies are reflected in the differing recommendations of international clinical nutrition guidelines [1,8,9,17]. However, the issue of enteral nutrition in ICUs is not insurmountable [18], as proper nutritional management can have a positive impact on patient outcomes [19]. Early mobilization combined with early nutrition exerted a notable impact on ICU length of stay, functionality, and quality of life [20]. Key areas for future research include the precise evaluation of patients’ nutritional status using validated tools, alongside accurate estimations of their energy, protein, carbohydrate, lipid, and micronutrient needs throughout the period of observation [21]. Clinical outcomes affected by nutritional support, such as quality of life, physical strength, and the incidence of post-intensive care syndrome, should be assessed using standardized methods to further enhance care for critically ill patients [17].

In conclusion, the present Special Issue presents personalized and dynamic approaches to nutrition, incorporating metabolic assessments and adjustments based on biomarkers to improve outcomes for critically ill patients. Undoubtedly, future research will deepen our knowledge of this crucial nutrition issue.
